# Review of Polymer-Based Composites for Electromagnetic Shielding Application

**DOI:** 10.3390/molecules28155628

**Published:** 2023-07-25

**Authors:** Yuqi Wang, Wei Zhao, Linli Tan, Yingru Li, Liu Qin, Shidong Li

**Affiliations:** 1 College of Intelligent Systems Science and Engineering, Hubei Minzu University, Enshi 445000, China; 2 Key Laboratory of Green Manufacturing of Super-Light Elastomer Materials of State Ethnic Affairs Commission, Hubei Minzu University, Enshi 445000, China; 3 Ningbo GMF New Material Technology Co., Ltd., Cixi 315300, China

**Keywords:** conductive polymer composites, electromagnetic shielding, uniform structure, foam structure, segregated structure, layered structure

## Abstract

The rapid advancement of electronic communication technology has greatly aided human productivity and quality of life, but it has also resulted in significant electromagnetic pollution issues. Traditional metals and alloys are often used for electromagnetic interference (EMI) shielding due to their excellent electrical conductivity. However, they have drawbacks such as being heavy, expensive, and having low corrosion resistance, which limits their application in electromagnetic shielding. Therefore, it is crucial to develop novel EMI shielding materials. Polymers, being highly flexible, corrosion-resistant, and possessing high specific strength, are frequently employed in electromagnetic shielding materials. In this review, we firstly introduce the basic theory of electromagnetic shielding. Then, we outline the processing methods and recent developments of polymer-based electromagnetic shielding composites, including uniform-, foam-, layered-, and segregated structures. Lastly, we present the challenges and prospects for the field, aiming to provide direction and inspiration for the study of polymer-based electromagnetic shielding composite materials.

## 1. Introduction

With the rapid advancement of electronic communications and wearable technology in the 5G era [1,2,3], people’s daily lives and productivity have been greatly enhanced. However, this technological progress has also led to a new environmental challenge—electromagnetic radiation pollution, which has raised significant concerns [4,5,6]. Alongside air pollution, noise pollution, and water pollution, electromagnetic radiation pollution has emerged as another major environmental issue [7,8]. Numerous studies have demonstrated that electromagnetic radiation not only disrupts the normal functioning and reduces the longevity of electronic devices but also poses health risks, including the development of various diseases [9,10]. Therefore, whether it is to maintain the normal operation of electronic devices or to protect human health, the study of electromagnetic shielding materials has become a focus of research in recent years [11,12,13]. Metals were initially employed as electromagnetic shielding materials due to their exceptional electrical conductivity [14,15,16], but their disadvantages are also obvious. First of all, metal materials have high density, are not easy to process, and have poor corrosion resistance. Secondly, metal materials mainly shield electromagnetic waves through reflection, which will inevitably cause secondary pollution [17,18,19,20]. These shortcomings limit the application of metal materials in electromagnetic shielding. High-entropy alloys (HEAs) are expected to break the limitations of traditional alloys in the field of electromagnetic shielding. HEAs have excellent mechanical properties, corrosion resistance, and oxidation resistance. These characteristics make HEAs effective electromagnetic shielding materials in extreme environments. In addition, since HEAs are composed of multiple elements, their lattice distortion will lead to a decrease in electrical conductivity, which may change the electromagnetic shielding mechanism of the material from reflection to absorption, thereby reducing the secondary pollution caused by reflection [21,22,23,24,25]. Polymers have attracted extensive attention due to their light weight, good processability, corrosion resistance, and low cost. Researchers are gradually developing polymers as electromagnetic shielding materials [26,27,28]. According to their components and composition, conductive polymer-based EMI shielding materials can be further separated into intrinsic and composite groups.

ICPs (Intrinsically Conductive Polymers) are a class of polymer materials with conjugated π bonds. They include PA (polyacetylene), PANI (polyaniline), PT (polythiophene), and PPy (polypyrrole). After doping, carriers are generated between polymer chains, making them conductive. These materials possess high conductivity, as well as the advantages of light weight, corrosion resistance, and good flexibility of polymer materials [29,30]. However, achieving high conductivity often requires doping, which involves complex preparation processes and high costs. Consequently, they are primarily used in special situations, such as military applications [31]. In contrast, CPCs (Conductive Polymer Composites) offer more advantages. Although the polymers used in CPCs are not conductive, they can be blended with conductive fillers to prepare lightweight, corrosion-resistant, low-cost, and processable electromagnetic shielding materials, allowing for greater flexibility in design [32,33]. Nevertheless, the characteristics of CPCs also present challenges. Increasing the content of conductive filler is necessary to improve the electromagnetic interference shielding effectiveness (EMI SE) of CPCs. However, excessively high filler content can compromise the mechanical properties and processability, and increase production costs. [33,34,35]. To address these issues, researchers have focused on enhancing the structure of CPCs, including foam structures [36,37,38], separated structures [39,40], and layered structures [41,42,43]. These improved CPCs with enhanced structures exhibit superior electromagnetic shielding capabilities and have gained widespread application. Presently, research on CPCs has become a popular topic, especially with the rapid advancement of 5G communication technology and the potential future prospects of 6G technology. As a result, CPCs will play a significant role in the development of EMI shielding technology [44,45,46].

The review discusses the principle of electromagnetic shielding and provides a summary of the research progress in polymer-based electromagnetic shielding composites. It focuses on the structure, preparation methods, and applications of these composites. Furthermore, the article explores the future development prospects of polymer-based EMI materials, aiming to provide valuable insights for professionals in relevant industries.

## 2. Electromagnetic Shielding Mechanism

**Figure 1:**
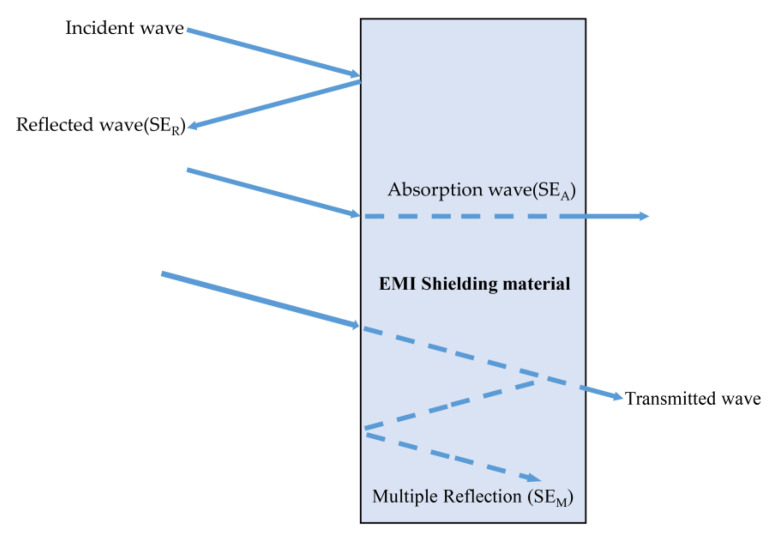
Electromagnetic shielding mechanism diagram.

The principle of electromagnetic interference (EMI) shielding refers to the ability of EMI materials to absorb, reflect, or weaken electromagnetic waves [47]. There are several theoretical explanations for the electromagnetic shielding process, including the eddy current effect theory, the electromagnetic field theory, and the transmission line theory. The transmission line theory is widely recognized due to its simplicity in calculation, high precision, and ease of understanding. Figure 1 illustrates the specific mechanism [48]. When an electromagnetic wave travels through a material, a portion of the wave is reflected on the surface of the electromagnetic shielding material caused by a discontinuity in the interface impedance (i.e., reflection loss efficiency, SE_R_). A part of the electromagnetic wave enters the material and is continuously attenuated due to loss (i.e., absorption loss efficiency, SE_A_). A portion of the electromagnetic wave will be dissipated by multiple reflections inside the material (i.e., multiple reflection efficiency, SE_M_), and the remaining electromagnetic waves will be transmitted in waves after passing through the shielding material. The shielding performance of a material is usually expressed by the shielding efficiency (SE), which can be expressed by Equation (1) [49,50,51,52].

SE = SE_R_ + SE_A_ + SE_M_ (1)

EMI SE is strongly associated with the charge, current, and polarization events occurring on the surface and inside the shielding enclosure [53,54]. When an electromagnetic wave is reflected and absorbed by the shielding surface, there is poor impedance matching between the shielding material surface and the free interface, resulting in induced charges in the magnetic field within the shielding material. Therefore, it is necessary for the shielding material to have good conductivity [55,56]. Conductivity is a crucial factor influencing the electromagnetic shielding ability of materials, since higher conductivity generates a larger number of free charges, leading to impedance mismatch and increased reflections. Increased reflection results in a higher SE_R_ of the material, subsequently increasing the SE of the material as well [57,58]. When an un-reflected electromagnetic wave enters the shielding material and is absorbed and attenuated, the material contains numerous dipoles that experience orientation polarization within the magnetic field. Hence, the shielding material must possess good magnetic conductivity, excellent electromagnetic loss, and an appropriate dielectric constant [59,60,61,62]; when the remaining electromagnetic wave reaches the transmission edge of the electromagnetic shielding enclosure, it undergoes multiple reflection attenuation. Shielding materials with a porous structure and a large number of interfaces can improve the frequency of multiple reflections and scatterings, thereby effectively enhancing the SE of the materials [63,64,65]. Consequently, an effective electromagnetic shielding material should exhibit both strong reflectivity and efficient electromagnetic wave absorption [66,67].

## 3. Research Progress of Polymer-Based Composites with Different Structures in the Field of Electromagnetic Shielding

Polymer-based EMI shielding materials have gained significant attention as a viable alternative to traditional metal materials. This is because conventional metals lack corrosion resistance and possess drawbacks such as difficult processing, heavy weight, poor air permeability, high price, and limited control over shielding effectiveness. Consequently, the use of traditional metals is restricted within certain applications [68,69]. Polymer-based materials offer several advantages, including lightweight properties, corrosion resistance, and ease of manufacturing. Moreover, for CPCs, an effective structure (conductive network) provides exceptional conductivity and ultra-high EMI shielding effectiveness. The prevailing polymer-based EMI shielding composites encompass uniform structure, isolation structure, porous structure, and layered structure [33,34,35].

### 3.1. Uniform Structure

The uniform structure refers to the dispersion of conductive filler in the matrix in a uniform manner. Common methods for preparing EMI shielding materials with such a structure include solution and melt blending, as well as in situ polymerization. These methods are preferred due to their low cost and simplicity. However, this type of material has a significant drawback. To enhance the EMI shielding effectiveness (SE), it is necessary to increase the filler content in the matrix. This, in turn, leads to a high percolation value of the material, ultimately impacting its mechanical properties [70,71,72,73]. Acharya et al. [74] prepared PVDF(polyvinylidene fluoride)/RGO(reduced graphene oxide) electromagnetic shielding composite; when the concentration of the material was 21%, the EMI SE reached 60 dB. Li et al. [75] obtained a POM(polyformaldehyde)/MWCNT(multi-carbon nanotube) composite. The composite, with a concentration of 40%, had an EMI SE of 70 dB. They observed that the electromagnetic interference shielding effectiveness of the material was influenced by the filler content. As the filler content increased in the matrix, the EMI SE of the material improved; however, this enhancement was accompanied by a reduction in its mechanical performance [51,74]. Consequently, it was crucial to modify the filler and develop a novel process to reduce the filler content [76]. Researches on poly-based electromagnetic shielding composites with uniform structure are summarized in Table 1. It is worth mentioning that the frequency range of commonly used electromagnetic waves is 0 to 400 Ghz. Most of the studies on the electromagnetic shielding performance of the polymer matrix composites mentioned in this article were carried out in the frequency range of 8 ∓ 12 Ghz (X-band). This preference is due to the X-band’s low atmospheric attenuation rate, where gas molecules and suspended particles in the atmosphere absorb and scatter electromagnetic waves, resulting in energy attenuation. As a result, the cost of transmitting electromagnetic waves in the X-band is relatively low while maintaining a high throughput. Consequently, the X-band is widely suitable for communication satellites, aviation and marine radars, and military applications [18,77,78].

#### 3.1.1. Solution-Blending Method

The production process of this method can be summarized as follows: Firstly, a suitable solvent is chosen to uniformly disperse or dissolve the conductive filler and the matrix. Next, the solvent is removed, and finally, a uniformly structured electromagnetic shielding material is prepared. Additionally, physical methods such as mechanical agitation or ultrasound are commonly employed in this process [92,98]. Vineeta et al. [83] prepared RGO/PdNi(palladium-nickel)/EVA(ethylene-vinyl acetate copolymer) composite through the solution-blending method. Meher et al. [73] employed the solution-mixing method to blend PANI and PVDF, resulting in a high-quality electromagnetic shielding composite material with a high dielectric constant and a low band gap for light energy. Zhang et al. [87] obtained PMMA(polymethyl methacrylate)/GNP(graphene nanoplatelet)-MWCNT composite via the solution-blending method. They found that the shielding properties of the material increased with an increase in fill content and that the EMI SE of composite with high aspect ratio was higher [73,83,87]. Shakir et al. [71]. used PS(polystyrene) as the matrix and PANI as the conductive filler to prepare PS/PANI composite. They verified the EMI SE of the PS/PANI blend film in the microwave and near-infrared regions. The experimental results proved that the blend had excellent EMI SE in the 9Ghz to 18Ghz band, the measured shielding effect was 45 dB, and the transmittance in the near-infrared region was <1%. Ankur et al. [94]. utilized CB(carbon black) as a nano-filler to fabricate blend composites of EMA (ethylene-co-methyl acrylate) and TPO (thermoplastic polyolefin) through solution blending. At a filler content of 30%, the EMI SE reached 29 dB, exceeding the 15 dB threshold required for commercial applications.

The method requires material compatibility with the solvent; otherwise, the material will not dissolve. Additionally, when using this method to prepare composite materials, the solution will contain a significant amount of solvents that are challenging to completely remove. The presence of these remaining solvents can affect the material’s mechanical properties and potentially harm the environment through volatilization and release into the atmosphere [99,100].

#### 3.1.2. Melt Blending

**Figure 2:**
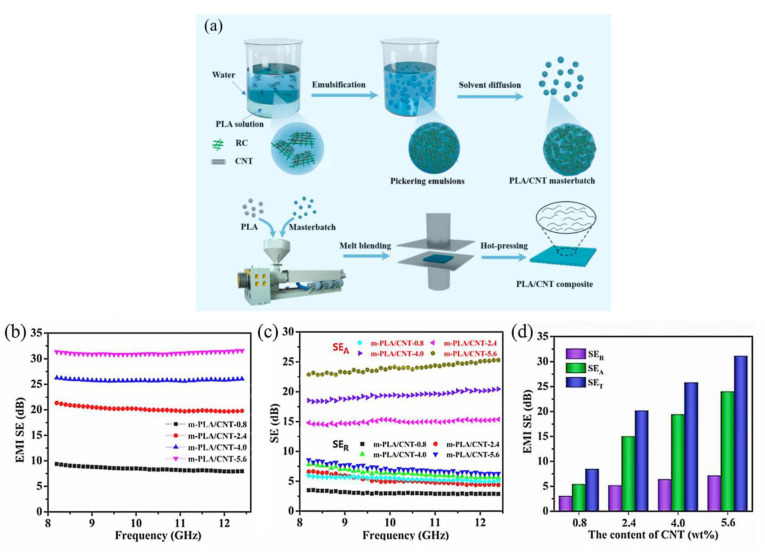
(**a**) The creation method of PLA/CNT compounds utilizing a “two-step dispersion strategy”; (**b**) the EMI SE, (**c**,**d**) in the X-band region; composites SE_A_, SE_R_, and SE_T_ [95].

This is the most frequent approach for producing uniformly structured polymer-based EMI shielding composites. It offers the advantages of being cost-effective and easily processed. In addition, the method is simpler to prepare than the solution-blending method, since no solvent is added to reduce the stickiness of the composites, which leads to the mass production of shielding materials using this method. The preparation process of this method involves pre-mixing the polymer and the filler initially, followed by uniform mixing above the melting flow temperature of the polymer matrix, and finally cooling to obtain the desired polymer-based composite material [101,102,103]. Li et al. [51] prepared two composites, POM/MWCNT and POM/GNP, using melt blending. Their preparation is simple and straightforward and the EMI SE of the composites was higher than commercial requirements. Zeng et al. [86] obtained PLA(polylactic acid)/PEO(polyethylene oxide)/GNP composite through the melt-blending method. Schmitz et al. [91] prepared ABS(acrylonitrile butadiene styrene copolymers)/CB/CNT(carbon nanotube) composite using melt blending. Their research found that increasing the filler led to an increase in conductivity and EMI SE. In addition, they had better physical properties [86,91]. Chen et al. [97] created TPU(thermoplastic polyurethane)/SiAPP(silicon-wrapped ammonium polyphosphate)/SCF(short carbon fiber)/Ti_3_C_2_Tx MXene composite with multiple structures. The findings demonstrate that the average EMI SE of the material reached 50.5 dB after adding Ti_3_C_2_Tx MXene into the TPU/SiAPP/SCF/composite. This is mainly due to the excellent electrical conductivity of MXene. Aiming at the problem that the current melt blending technology has difficulty endowing polyacrylonitrile with high conductivity, Wang et al. [95] proposed a “two-step dispersion strategy” method, i.e., creating PLA/CNT composite materials using the Pickering emulsion template and masterbatch mixing as a design framework. The results show that the PLA/CNT composite had a low permeation threshold (0.46 vol%) and high electrical conductivity (72.2 S/m) at 5.6% CNT loading. The performance of the material prepared by this method was better than that of the PLA/CNT composite obtained by straight melt mixing. In addition, the composites had an ideal EMI SE (31.1 dB), as shown in Figure 2.

The melt-blending method offers several advantages, including efficient processing, affordability, environmental friendliness, and versatile applications. Consequently, it has found widespread use in both production and experimentation. Nonetheless, the significant shear stress generated during the manufacturing process can damage the filler’s structure, reduce performance, and ultimately compromise the composite material’s electromagnetic interference (EMI) shielding capability [104,105].

#### 3.1.3. In Situ Polymerization Method

**Figure 3:**
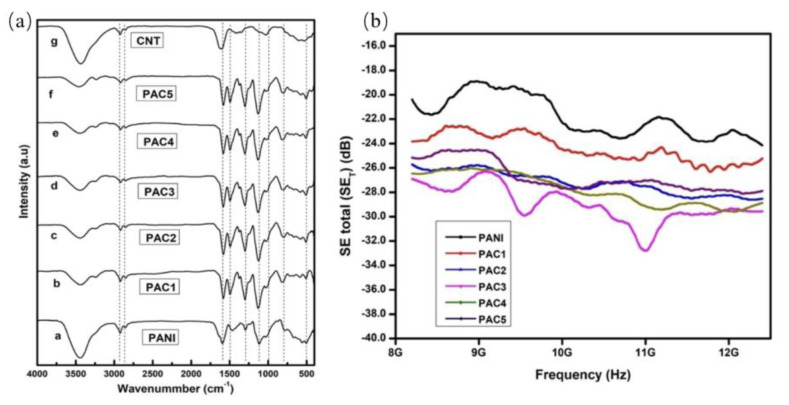
(**a**) a−g SWCNT, PANI, and PANI-SWCNT composites’ FTIR spectra; (**b**) PANI + SWCNT composites’ total shielding efficiency (SET) at ambient temperature [96].

This method involves mixing the conducting filler with the polymer thoroughly prior to polymerization. Low shear force is applied through mechanical stirring during the polymerization process to achieve an even distribution of the conductive filler within the polymer matrix. This ultimately yields a polymer-based electromagnetic shielding material with a uniform structure. In contrast to the solution method and the melting method, this approach demonstrates superior dispersion capabilities while minimizing damage to the filler structure. It has also been widely utilized in practical production and experimentation [106]. Mohsina et al. [82] prepared PEDOT(poly-3,4-ethylenedioxythiophene)/GNP composite using an in situ polymerization method. They observed that absorption was the main shielding mechanism of the material, and that PEDOT could absorb more than 90% of electromagnetic waves. Zhang et al. [93] also prepared hetero-structured composite with this method. They found that the heterostructured composite had better EMI SE. This is mainly because of the synergistic effect of interfacial polarization, electrical conductivity, skin depth effect, and heterostructure. Raju et al. [96] synthesized PANI and SWCNT(single-walled carbon nanotube) nanocomposites using the ultrasonic method and in situ polymerization technology. As shown in Figure 3a, the chains of polymers and carbon nanotubes interacted more effectively, and the energy band positions at 1577, 1456, and 1103 cm^−1^ were slightly changed and the intensity was increased compared with pure PANI and SWCNT, which indicates a p-p interaction between the PANI ring and the graphitic structure of SWCNTS. As shown in Figure 3b, the overall EMI SE improves as the SWCNTs content grows, since increasing the SWCNTs content considerably raises the charge of the carriers, boosting the conductivity of the nanocomposite. When the concentration of SWCNT in the matrix is 3%, the EMI SE value reaches 32.8 dB. However, when the SWCNT content is too high (more than 3 wt%), the total EMI SE declines, which could be due to a lack of network due to the hopping transport mechanism, which impacts carrier transport, conductivity, and SE.

The viscosity of the system gradually increases during the process of preparing composite materials using this method. Despite the minimal shear force created by mechanical stirring, it sufficiently promotes the uniform and sustained distribution of conductive fillers, without altering the filler structure. However, due to the conductive filler’s interfacial contact with the polymer matrix, it is often necessary to modify the filler’s surface to achieve good dispersion, which affects the filler’s physical and chemical properties [107,108,109].

### 3.2. Foam Structure

Shielding materials with foam structures offer numerous advantages, including anti-aging, corrosion resistance, light weight, and low cost. These materials find applications in various fields, such as aerospace, communication electronics, and military engineering [110,111,112,113]. Compared to other materials, foam-structured EMI shielding composites excel in using a small amount of conductive filler to achieve higher electromagnetic shielding performance, resulting in lower permeability values. Moreover, the hole walls on the surface provide multiple interfaces, allowing for multiple reflections and absorptions of electromagnetic radiation within the material. This property enhances the material’s EMI SE and reduces pollution caused by surface reflection to some extent. There are three primary processes used to create foam structures: physical foaming, chemical foaming, and freeze-drying [114,115,116]. Table 2 shows the recent research progress of foam-structured electromagnetic shielding materials.

#### 3.2.1. Physical Foaming Method

Currently, there are two primary methods of physical foaming. The first method involves fully dissolving the inert gas into the material in an inert gas environment at a specific temperature and pressure, followed by depressurization to form pores inside the material for foaming. The second method utilizes the evaporation and vaporization of a low-boiling liquid within the material through increased temperature to achieve foaming. Among these methods, the supercritical CO_2_ (sc-CO_2_) foaming method is the most widely used due to its operability and sustainability [134]. Wang et al. [123] obtained lightweight TPU/MWCNT composite with the sc-CO_2_ foaming method. Zou et al. [125] combined the sc-CO_2_ foaming method and annealing treatment to prepare PS/PMMA/MWCNT foam composites with a low percolation threshold. Luo et al. [127] prepared a PBS(poly-butylene succinate)/CNT composite with this method. Their research proves that low-cost, degradable, and good EMI SE composites can be prepared by supercritical carbon dioxide foaming. Bai et al. [132] prepared PVDF/GNPs/CNTs/Ni nanocomposite foam materials using sc-CO_2_ foaming technology. CNTs and GNPs can enhance the dielectric loss of PVDF composites, whereas Ni can increase the magnetic loss of PVDF composites. Porous structures can also be used to provide multi-interface reflection attenuation. The formation of a nanofiller–nanofiller network in PVDF composite foam raises its dielectric properties and EMI SE (19.4 dB). In another experiment, Bai et al. [133]. obtained ABS/CNTs foam composite prepared with the sc-CO_2_ method. Compared with pure ABS, the storage modulus and complex viscosity of ABS/CNTs composites were improved by nearly two orders of magnitude, and the electrical conductivity was increased by about nine orders of magnitude. The EMI ratio shielding performance of ABS/CNTs foam improved from 22.75 to 26.6 dB at 145 °C compared to the un-foamed sample. They found that for various ABS foam materials with the same carbon nanotube concentration, the greater the cell size, the larger the specific interface area, and the better the EMI shielding performance.

The physical foaming method is cost-effective, particularly concerning the expenses of CO_2_ and N_2_. However, the current research primarily concentrates on the preparation of general-purpose plastics. There is a considerable amount of progress required to apply this technology to engineering plastics.

#### 3.2.2. Chemical Foaming Method

**Figure 4:**
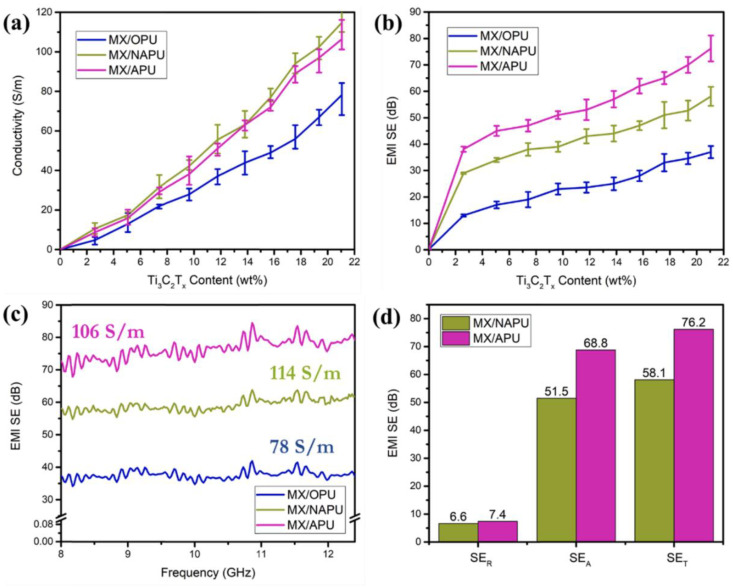
(**a**) MX/PU composite foams’ electrical conductivities; (**b**) EMI SEs of MX/PU mixed foams with varying Ti_3_C_2_Tx compositions; (**c**) MX/PU mixed foams with EMI SEs operating in the X-band; (**d**) MX/NAPU and MX/APU’s average EMI SE_T_, SE_R_, and SE_A_ in the X-band [128].

The chemical foaming method involves heating the foaming agent to produce gas, generating cells inside the composite material. Additionally, the chemical reaction between the material’s components can be utilized for foaming. Common chemical blowing agents include sodium chloride, sodium bicarbonate, and nitroso compounds [135]. Some recent studies have used this method to prepare electromagnetic shielding composites [36,117,118,120,126]. Zhu et al. [36] used chemical injection foaming to create a high-strength and lightweight PA6(polyamide 6)/CF(carbon fiber) composite foam. The inclusion of CF enhances the viscoelasticity, melt strength, and foaming ability of PA6/CF composites. The PA6/CF composite foam exhibits the greatest foaming effect and the maximum EMI shielding performance (36.6 dB) when the CF component is 22 wt%. PA6/CF composite foam has higher electromagnetic shielding performance than solid foam, indicating that the cell structure of PA6/CF composite foam helps to improve EMI SE. In addition, the tensile strength, flexural strength, and flexural modulus of PA6/CF composite foam rose as CF content was increased and cell structure was improved. Kim et al. [128]. prepared a MXene/APU(auxetic polyurethane) composite foam. Since APU has a larger specific surface area, this will add another conductive interface, allowing electromagnetic waves to scatter through the skin effect, resulting in a longer propagation distance for electromagnetic waves before transmission, allowing the material to capture more electromagnetic waves and exhibit better EMI SE. The results are shown in Figure 4. Under the same density and electrical conductivity, the EMI shielding efficacy of MXene/APU composite material is 31.2% higher than other similar products, reaching 76.2 dB.

The status of the chemical foaming method closely resembles that of the physical foaming method. It finds primary application in the production of general-purpose plastics and is infrequently employed in the field of engineering plastics.

#### 3.2.3. Freeze-Drying Method

**Figure 5:**
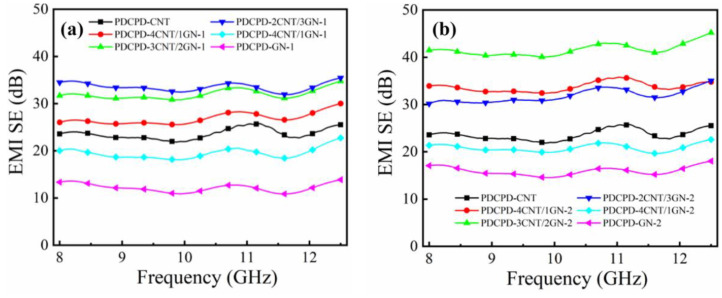
EMI SE of (**a**) PDCPDCNT/GN-1, (**b**) PDCPD-CNT/GN-2 composites [130].

The freeze-drying process is used to control the formation of ice crystals while constructing cell shape on the micro/nano scale. Due to the advantage of controllable multi-scale ordered structure, this method has become an effective means of designing high-performance foam-structured polymer-based EMI shielding materials [136,137]. MWCNT and MCHM (mesoporous carbon hollow microsphere) were employed as conductive fillers, while WPU(water-based polyurethane)/PVA served as the matrix. By regulating the moisture content and freeze-drying, Liang et al. [129] created foam composites. The outcomes demonstrate that changing the density can alter the foam composite’s EMI SE, and the synergistic effect of hollow mesoporous carbon and multi-walled carbon nanotubes can enhance the EMI SE of the composite. When the density was 232.8042 mg cm^−3^ and MWCNT/WPU was 40 wt%, the electrical conductivity of the composite was 30.2 S m^−1^, and the EMI shielding efficiency was 23 dB. Wang et al. [130] obtained PDCPD(poly dicyclopentadiene)-CNT/GN EMI shielding composite material with a porous structure. They found that increasing the diameter of the GN in the composite could form a more regular and denser conductive network structure, which makes the material have higher electromagnetic EMI SE. As shown in Figure 5, it could reach 43 dB in the X-band.

To further broaden the application of the freeze-drying method, two challenges need to be addressed. First, it is essential to control ice crystal formation to achieve a suitable structure. Second, this method is applicable only to systems that are soluble or decomposable in water and imposes specific criteria for selecting polymers and fillers [138,139].

### 3.3. Segregated Structure

The polymer itself has low electrical conductivity, necessitating the continuous addition of conductive fillers to the polymer matrix to impart conductivity. However, excessively high filler content increases costs and processing difficulty, and damages the material’s mechanical properties [140,141,142]. These challenges are effectively addressed by creating polymer-based EMI shielding materials with segregated structures. The isolation structure of these materials ensures that conductive fillers are distributed only between the polymer particle interfaces, preventing them from dispersing freely throughout the matrix. This restricted distribution increases the probability of filler overlap at the interfaces and significantly reduces the percolation threshold. Consequently, even at low filler content, the composite material exhibits strong electrical conductivity and EMI shielding capability [143,144,145]. Research by numerous investigators has demonstrated that the generation of a segregated structure is advantageous for reducing the percolation threshold of the composite, while synergistic filler interactions enhance the material’s shielding ability and mechanical properties [39,40,146,147,148,149]. The recent studies of segregated-structured electromagnetic shielding materials are summarized in Table 3. Zhang et al. [150] prepared PVDF/Fe_3_O_4_-RGC composite using layered electrostatic assembly and hot pressing. Wu et al. [151] obtained Fe_3_O_4_@PA6/MWCNT composite with layer-adding manufacturing and molding. They found that establishing a separation structure could form a complete conductive network, and adjusting the content of magnetic fillers could effectively improve the EMI SE of the composite. Yang et al. [152] obtained TPU/EG(expanded graphite)/AG(silver) composite material. The inclusion of the Ag component at just 0.58 vol% led to remarkable improvements in the syntactic foam’s conductivity and EMI SE, reaching 171.2 S/m and 56.3 dB, respectively—well exceeding the minimum requirements for commercial EMI shielding. The introduction of a porous structure effectively mitigates the impedance mismatch between the surrounding air and the composite material, facilitating more incident electromagnetic waves and reducing reflectivity. The material’s reflection efficiency in the X-band decreased from 99% to 31%, successfully mitigating secondary electromagnetic wave emission pollution. Additionally, the strong interfacial bonding between the filler and matrix ensures excellent compressibility recovery and shielding stability, even after 50 compression cycles. Wang et al. [153].prepared BPEI(multiblock polyetherimide)/MLG(multilayer graphene) composites. Due to the existence of two blocks with large TG differences in BPEI, the polymer has a high chain mobility, which allows it to inter-diffuse between the BPEI particle interfaces below TK-N, forming a good isolated conductive network. The findings demonstrated that the BPEI/MLG composite loaded with 5.0 wt% MLG generated an isolation structure at a temperature of 270 °C, thereby giving it an electrical conductivity of 313.5 S/m and an EMI shielding performance of 62.2 dB. Ma et al. [154] prepared a CNT/PDMS(polydimethylsiloxane) composite by establishing chemical bonds between them. The cross-linked network of PDMS microspheres prevents the penetration of CNTs into the polymer microcells, resulting in the formation of segregated structures. The results showed that even with only 2.2 vol% of CNT, this composite exhibited exceptional shielding performance at 47.0 dB. Notably, the tensile strength and elongation at break of the composite material reached 3.6 MPa and 87.0%, respectively, marking a substantial increase of 35.0 and 7.0 times compared to traditional isolation composite materials. Additionally, the material exhibited excellent reliability, retaining a high EMI SE of 80% even after undergoing 1000 strain-release cycles at 30% ultimate elongation.

Materials with a segregated structure have garnered considerable interest due to their outstanding EMI shielding performance and remarkably low percolation threshold. However, the distribution of conductive fillers at the polymer particle interface poses challenges, hindering molecular chain diffusion and affecting interface compatibility. Additionally, agglomerated conductive fillers generate micropores along the direction of the conductive path. These adverse circumstances will impact the mechanical characteristics of the polymer-based EMI shielding material with a segregated structure [155,156,157,158]. Yang et al. [159] added SiO_2_ particles to PDMS, and the EMI SE of the composite was improved due to the volume exclusion effect and multi-interface. However, nanoparticles disrupt conductive networks.

**Table 3:** Summary of the research on poly-based electromagnetic shielding composites with segregated structure.

Materials *	Filler Loading	EMI SE (dB)	Thickness (mm)	Frequency	Ref.
Starch/CNT	3 vol%	33.12	1.6	8–12 Ghz	[146]
PEBA/CNS composites	0.2 wt%	33	3.4	8–12 Ghz	[39]
PS/CNT/PEDOT:PSS	6 wt%	33.4	0.6	8–12 Ghz	[147]
PVDF/Fe_3_O_4_-RGC	2.02 vol%	44.5	-	8–12 Ghz	[150]
Fe_3_O_4_@PA6/MWCNT	9 wt%	24.8	0.5	8–12 Ghz	[151]
BPEI/MLG	5 wt%	62.2	2.3	8–12 Ghz	[153]
PP/RGO/MWCNT	5 wt%	16	-	8–12 Ghz	[145]
PLA/PBS/MWCNT	2 wt%	27.56	2	8–12 Ghz	[148]
PA12/CNT	5.66 wt%	23.9	2	8–12 Ghz	[149]
POM/CNT	4 wt%	21.5	2	8–12 Ghz	[40]
BN/GNP/PPS	40 wt%	70	3	8–12 Ghz	[157]
SiO_2_/CNT/PDMS	35.4 vol%	52.2	2	8–12 Ghz	[159]
NR/CNT	7 wt%	44.2	2	8–12 Ghz	[158]
PBS/CNT	2 wt%	24	2	8–12 Ghz	[142]
CNT/PDMS	2.2 vol%	47	2	8–12 Ghz	[154]
TPU/EG/AG	0.58 vol%	56.3	-	8–12 Ghz	[152]

* PEBA—polyether block amide; CNS—carbon nanostructures; PSS—polystyrene sulfonate; RGC—reduced graphene oxide/single-wall carbon nanotube; PP—polypropylene; BN—boron nitride nanosheet; PPS—polyphenylene sulfide; NR—natural rubber; EG—expanded graphite.

### 3.4. Layered Structure

**Figure 6:**
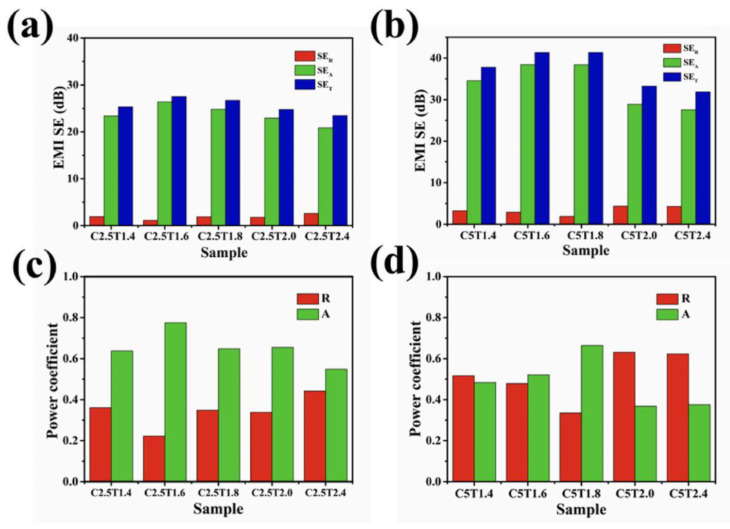
(**a**,**b**) Average electromagnetic interference shielding efficiency; (**c**,**d**) TPU/MWCNT sandwich-structured composites’ and foams’ coefficient of power [173].

The polymer-based EMI shielding composite material with a layered structure contains multiple interfaces, allowing for repeated reflection of electromagnetic radiation, leading to polarization loss and electromagnetic wave absorption loss [160,161]. This layered structure of EMI shielding material can achieve high electromagnetic shielding performance even with a low content of conductive filler, while offering design flexibility. Typically, polymer-based electromagnetic shielding composites with a layered structure exhibit sandwich and gradient configurations, which can be easily prepared using methods like vacuum filtration and deposition [162,163,164]. Due to their strong absorbing ability, these layered materials find extensive applications in critical fields such as military technology and precision instruments, significantly reducing secondary pollution caused by electromagnetic wave reflection [165,166,167,168]. Yu et al. [169] prepared PI(polyimide)/Ti_3_C_2_Tx Mxene/AgNW(silver nanowire) composite through electrostatic spinning and a hot-press approach. Wu et al. [170] obtained PPA6@NiM(magnetic PA6 microspheres)/PDMS composite using the solvent coprecipitation method. Chu et al. [171] prepared h-PANI/CNF/Mxene composite films with the alternating vacuum-assisted filtration method. Guo et al. [172] obtained ANF(aramid nanofiber)/AgNW/GN composite films through a vacuum filtration and hot-press approach. They found that the electromagnetic shielding material with a layered structure exhibited a strong absorbing ability, primarily attributed to the formation of a dense conductive network in the core layer of the material and excellent adhesion between the layers [42,169,170,171,172]. Table 4 shows the recent research of layered-structured electromagnetic shielding materials. Song et al. [173] obtained TPU/MWCNT electromagnetic shielding material with a sandwich structure through the CO_2_ foaming method. Figure 6 shows that at 2.5% MWCNT content, the average EMI SE exceeded 20 dB (meeting the lowest commercial standard). With 5% MWCNT content, the average EMI SE surpassed 30 dB (shielding over 99% of electromagnetic waves), and achieved maximum SE and absorbance of 53.3 dB and 0.66, respectively. This is attributed to the Fabry–Perot resonance effect, causing attenuation of electromagnetic waves in the electromagnetic shielding material with a sandwich structure and a certain thickness of the middle layer, thereby demonstrating excellent electromagnetic shielding performance.

Liu et al. [174] prepared NiFe_2_O_4_/AgNW/EPM(expandable polymer microsphere) composite through the vacuum filtration and hot-pressing method. The resulting asymmetric foam allows electromagnetic radiation to follow the “absorption-reflection-absorption” path, facilitated by the magnetic NiFe_2_O_4_/EPM impedance matching layer and the conducting AgNW/EPM shielding layer. The findings demonstrate that the 2 mm thick 10-AgE-10-NFOE foam outperformed other known shielding materials with a remarkable EMI SE of 66.5 dB, an exceptionally low conjugate reflection coefficient of 0.15, and a green shielding index (gs) of 5.67. Additionally, the material exhibited a thermal conductivity of 0.028 W/(m·K), comparable to that of air, signifying good thermal insulation performance. Moreover, the electromagnetic shielding performance remained at 81.2% even after 1000 compression cycles. Xing et al. [175]. obtained MXene/AgNW/MoS_2_ composite films by vacuum filtration and atomic deposition. The composite film achieves an exceptional maximum shielding value of 86.3 dB in the X-band, despite its mere 0.03 mm thickness. This impressive performance was attributed to the three-dimensional conductive network structure formed by the AgNW skeleton interspersed in the MXene sheet, significantly enhancing conductivity. Furthermore, the presence of a heterogeneous interface between the MXene/AgNW and AgNW layers contributed to an amplified multilayer reflection loss of electromagnetic waves.

Polymer-based electromagnetic shielding composite materials with a layered structure offer significant design freedom, low cost, and strong microwave absorption ability. However, they encounter challenges in actual production [41,176,177]. The bonding of the layers in the layered structure material using physical methods can degrade the compatibility between the layers and may also lead to cracks forming during the bonding process. Consequently, these drawbacks have the potential to restrict the application of multilayer polymer-based electromagnetic shielding materials [43,178].

**Table 4:** Summary of the research on poly-based electromagnetic shielding composites with layered structure.

Materials *	Filler Loading	EMI SE (dB)	Thickness (mm)	Frequency	Ref.
PI/Ti3C2Tx Mxene/AgNW	20 wt%	79.54	0.15	8–12 Ghz	[169]
PLA/MWCNT	5 vol%	26	1.5	24–40 Ghz	[41]
PPA6@NiM/PDMS	54.9 wt%	39.9	1	8–12 Ghz	[170]
PLLA/GNP/Fe_3_O_4_	10wt%	41.7	0.4	8–12 Ghz	[176]
CF@(CNT/Fe_3_O_4_/EP)	0.045 wt%	30.5	2	8–12 Ghz	[177]
h-PANI/CNF/Mxene	8 wt%	35.3	-	8–12 Ghz	[171]
PVDF/SiBi_58_/Co-C	30 vol%	50	2	8–12 Ghz	[166]
EP/LMPA	20 vol%	20	-	8–12 Ghz	[161]
SR/Mxene/Fe_3_O_4_	21.2 wt%	55.5	2	8–12 Ghz	[165]
CF/GF/PDMS	1 wt%	30	1	8–18 Ghz	[167]
ANF/AgNW/GN	2 vol%	68.3	0.04	8–12 Ghz	[172]
NR/MXene/CNT	50 wt%	49.9	0.2	8–12 Ghz	[43]
PEEK/MWCNT	20 wt%	44.5	0.56	8–12 Ghz	[42]
TPU/MWCNT	5 wt%	53.3	2.4	8–12 Ghz	[173]
NiFe_2_O_4_/AgNW/EPM	10 wt%	66.5	2	8–12 Ghz	[174]
MXene/AgNW/MoS_2_	10 wt%	86.3	0.03	8–12 Ghz	[175]

* LMPA—low-melting-point alloy; SR—silicone rubber; GF—graphene fiber; PEEK—polyether-ether-ketone.

## 4. Applications of Polymer-Based Electromagnetic Shielding Composites

Electromagnetic waves are vital resources closely intertwined with people’s lives [67,179]. However, the issue of electromagnetic radiation has also caused significant challenges to both human health and the proper functioning of electronic equipment. In response, numerous devices with electromagnetic shielding capabilities have been developed. This paper centers on the exploration of polymer-based EMI shielding composites in both military and civilian applications.

### 4.1. EMI Shielding Composites in Military Fields

In the military field, EMI shielding composite materials are primarily employed as stealth materials, particularly in aircraft and tank stealth technology [180,181]. Radar, a device using radio waves for target tracking and location determination, operates based on the principle of emitting electromagnetic waves toward the target. Upon hitting the target, some electromagnetic waves are reflected back, and the radar analyzes the echo signal to calculate the target’s position [182]. Reducing electromagnetic wave reflection is crucial in modern warfare to prevent enemy detection of reflected electromagnetic waves and ensure the secrecy of military facilities [183]. There are two primary methods for achieving stealthiness in fighters: specially designing the aircraft’s shape to minimize electromagnetic wave reflection and using materials with wave-absorbing properties. Polymer-based electromagnetic shielding composites have gained increasing usage due to their excellent wave-absorbing ability, lightweight, and corrosion resistance [184]. Teber et al. [185] used PAN(polyacrylonitrile) as the matrix and added metal particles to it to prepare a light-weight radar absorbing material. Chen et al. [186] used SR(silicone rubber) as the matrix and RGO as the filler to prepare a composite material with good radar absorbing ability. In the frequency range of 4–18 Ghz, its absorption capacity could reach up to 37.8 dB. Tank stealth technology comprises camouflage and radar stealth. Camouflage involves using surface coatings and camouflage nets to achieve visual and infrared stealth for tanks. PU(polyurethane) plays a pivotal role in coatings due to its excellent adhesion [187]. Radar stealth means that tanks use wave-absorbing materials or special shapes to reduce electromagnetic wave reflections to achieve stealth effects. Vivek et al. [188] obtained γFe_2_O_3_/Ba_4_Co_2_Fe_36_O_60_-EP(epoxy resin) radar-absorbing composite. In the frequency range of 2–18 Ghz, its maximum absorption rate could reach 98.9%. In addition, studies have shown that materials such as PVC(polyvinyl chloride), PS, and PANI can also have good wave-absorbing capabilities through intelligent processing [189,190].

### 4.2. EMI Shielding Composites in Civilian Field

In the civilian field, polymer-based electromagnetic shielding composite materials find extensive applications in personal protection, building protection, and equipment protection. Long-term exposure to high-frequency electromagnetic radiation can severely damage people’s cardiovascular and nervous systems [191,192]. Therefore, designing clothing with excellent electromagnetic radiation protection ability offers an effective solution to this problem. PAN, PVA(polyvinyl alcohol), PVDF, and other polymers are widely utilized because they can be processed into conductive fabrics that are highly flexible, lightweight, and possess strong absorbing ability [193,194,195]. Guo et al. [196] prepared TaC(tantalum carbide)/PAN fibers with high strength and excellent flexibility. Its tensile strength can reach up to 9.5 MPa, and its EMI SE can reach 37.7 dB in the X-band. Long et al. [197] obtained Ag-decorated PVA/Fe_2_O_3_ nanofiber composites. On the X-band, it had an EMI SE maximum of 45.2 dB. In areas with relatively strong electromagnetic radiation, it is essential to implement appropriate protective measures against electromagnetic radiation pollution. Polymer-based electromagnetic shielding composite materials, known for their excellent wave-absorbing properties, are also employed as wave-absorbing materials in buildings [198,199]. Given the serious interference that electromagnetic waves can cause to electronic equipment in operation, providing electromagnetic protection for such equipment is highly essential. Polymer-based composites emerge as the most suitable protective materials owing to their high absorption, low reflection, broadband capability, lightweight, and high strength [200,201]. Tao et al. [202] prepared CNF(cellulose nanofiber)/MXene/MCHS(mesoporous carbon hollow spheres) composite films for electromagnetic shielding protection. Because MCHS has a porous structure, electromagnetic waves can be reflected multiple times in the pores, which effectively reduces the reflection of electromagnetic waves and effectively reduces the secondary pollution caused by reflection. High-precision equipment benefits from an ideal protection method that involves a combination of reflective and absorbing shielding coatings. Reflective coatings block the entry of external electromagnetic waves, while absorbing coatings neutralize the remaining electromagnetic waves, resulting in an effective electromagnetic shielding effect.

## 5. Conclusions and Outlook

Based on the above summary, it can be concluded that polymer-based electromagnetic shielding composites are effective for solving electromagnetic radiation and have been widely used. This review focuses on the latest research progress of polymer matrix composites in electromagnetic shielding. Composites with a uniform structure are simple to prepare and have a low cost. However, their EMI SE is generally not particularly high. Improved shielding performance means higher filler content, which may increase the manufacturing cost of the material and deteriorate its mechanical properties. For materials with a uniform structure, conductive fillers with high aspect ratios are commonly used at present, but future research should prioritize developing materials with low conductive filler content while ensuring electrical conductivity. Composite materials with foam structures are widely used in flexible sensors due to their light weight and low density. However, further studies are needed on their mechanical properties, primarily due to their reduced density during the foaming process, which leads to deteriorated mechanical properties due to higher porosity. In the future, adjusting the phase composition and filler distribution to develop lightweight, high-strength foam composites may become an important research direction. The conductive fillers of composites with isolated structures are only distributed between the interfaces of polymer particles, increasing the probability of conductive fillers overlapping at the interface and reducing the percolation threshold. This enables the material to have high conductivity and electromagnetic shielding performance even with low filler content. However, the distribution of conductive fillers at the interface hinders the diffusion of molecular chains between polymers, affecting interface compatibility, and leading to the formation of micro-holes due to gathered conductive fillers along the direction of the conductive path, often resulting in deteriorated mechanical properties. New preparation methods should be developed to address this issue. Layered composites can be designed flexibly, creating multiple functional layers with numerous interfaces that reflect electromagnetic waves multiple times, enhancing polarization loss and absorption loss to improve electromagnetic shielding effectiveness. However, deployment of layered structure polymer-based electromagnetic shielding composites is impacted by compatibility and layer cracking issues. Currently, this problem is often solved by adding an adhesive or introducing an interface-coupling agent, but these additives may pose environmental challenges. For future development, focus should be placed on improving existing additives, aiming for non-toxic, harmless, green, and environmentally friendly solutions. With the continuous development of wireless communication technology and micro-electronic devices, electromagnetic shielding materials with only a single function may not suffice for future needs. The trend for future development lies in lightweight, flexible, super-hydrophobic, ultra-thin, self-healing, corrosion-resistant, flame-retardant, high-strength, and other multi-functional electromagnetic shielding composite materials. 

## Figures and Tables

**Table 1:** Summary of the research on poly-based electromagnetic shielding composites with uniform structure.

Materials *	Filler Loading	EMI SE (dB)	Thickness (mm)	Frequency	Ref.
PVA/GN/Fe_3_O_4_	0.1 wt%	40.7	0.2	8–12 Ghz	[79]
EP/PES/MWCNT	2.9 wt%	23	2.2	8–12 Ghz	[80]
GN/CFN	20 wt%	73	2	8–12 Ghz	[72]
VGCNF/PVDF	5 wt%	16.4	0.82	30 Khz–1.5 Ghz	[81]
PEDOT/GNP	15 wt%	18	-	8–12 Ghz	[82]
RGO/PdNi/EVA	1 wt%	30	-	8–12 Ghz	[83]
POM/MWCNT	40 wt%	45.7	0.15	8–12 Ghz	[75]
PE/PVDF/Fe_3_O_4_/CNT	10 wt%	26	2.6	18–26 Ghz	[84]
PLLA/MWCNT	10 vol%	23	2.5	8–12 Ghz	[85]
PVDF/RGO	21 wt%	60	1.5	8–12 Ghz	[74]
PLA/PEO/GNP	6 wt%	10.5	-	8–12 Ghz	[86]
PMMA/GNP-MWCNT	8 wt%	36	2	8–12 Ghz	[87]
PDMS/GA	12.5 wt%	52	3	4–16 Ghz	[88]
PVDF/Ba_4_CO_2_Fe_36_O_60_	20 wt%	83	0.12	8–18 Ghz	[89]
CF/PAA/Fe_3_O_4_	10 wt%	40.6	3.5	2–18 Ghz	[90]
PVDF/PANI	30 wt%	65	1	8–12 Ghz	[73]
ABS/CB/CNT	3 wt%	29	2	8–12 Ghz	[91]
PP/MWCNT	20 wt%	47	2	8–12 Ghz	[92]
BF/PANI	7 wt%	35.73	-	8–12 Ghz	[93]
PS/PANI	40 wt%	45	0.25	8–12 Ghz	[71]
CB/EMA/TPO	30 wt%	29	1	14–20 Ghz	[94]
PLA/CNT	5.6 wt%	31.1	-	8–12 Ghz	[95]
SWCNT/PANI	3 wt%	32.8	0.5	8–12 Ghz	[96]
TPU/SiAPP/SCF/Ti_3_C_2_Tx MXene	20 wt%	50.5	1	8–12 Ghz	[97]

* GN—graphene; PVA—polyvinyl alcohol; CFN—carbon fiber network; VGCNF—vapor-grown carbon nanofiber; PE—polyethylene; PLLA—poly (l-lactic acid); PLA—polylactic acid; PEO—poly(ethylene oxide); GA—graphene aerogel; CF—carbon fiber; PAA—polyacrylic acid; PP—polypropylene; BF—Bagasse Fiber.

**Table 2:** Summary of the research on poly-based electromagnetic shielding composites with foam structure.

Materials *	Filler Loading	EMI SE (dB)	Thickness (mm)	Frequency	Ref.
TEG/PU	3 wt%	20.4	-	8–12 Ghz	[117]
EVA/PPy/AgNPs	52 wt%	107.45	2.4	8–12 Ghz	[118]
PEN/GN/CNT/Fe_4_O_3_	3.5 wt%	38	0.6	8–12 Ghz	[37]
TPU/Ti_3_C_2_Tx MXene	0.66 vol%	72.2	2	8–12 Ghz	[119]
CO_3_O_4_/CNT/MF	-	25.6	3	8–12 Ghz	[120]
PET/BAHT/SWCNT	2 wt%	45.7	-	8–12 Ghz	[121]
RGO/PU	5 wt%	23	-	8–12 Ghz	[122]
CMF/SiO_2_/CNT	30 wt%	61.34	2	8–12 Ghz	[38]
TPU/MWCNT	2.5 wt%	44.86	-	10–14 Ghz	[123]
PVDF/CNT	8 wt%	41	1	8–12 Ghz	[124]
PS/PMMA/MWCNT	2 vol%	25.3	2	8–12 Ghz	[125]
ABS/CB:CNT	15 wt%	81.3	5	8–12 Ghz	[126]
PBS/CNT	4 wt%	24	-	8–12 Ghz	[127]
PA6/CF	22 wt%	36.6	1	18–26 Ghz	[36]
MXene/APU	22 wt%	76.2	5	8–12 Ghz	[128]
MWCNT/WPU/PVA/MCHM	40 wt%	23	2.5	8–12 Ghz	[129]
PDCPD-CNT/GN	3.5 wt%	43	3	8–12 Ghz	[130]
PEI/Ti_3_C_2_Tx MXene/AG	1 wt%	28	-	8–12 Ghz	[131]
PVDF/GNPs/CNT/Ni	16 wt%	19.4	1	8–12 Ghz	[132]
ABS/CNT	7 wt%	26.6	2	8–12 Ghz	[133]

* TEG—thermally exfoliated graphene; PU—polyurethane; AgNPs—Ag nanoparticles; PEN—Poly(arylene ether nitrile); MF—melamine-formaldehyde; PET—poly(ethylene terephthalate); BAHT—bis(6-aminohexyl) terephthalamide; CMF—carbonated melamine foam; PBS—poly (butylene succinate); PDCPD—polydicyclopentadiene.

## Data Availability

No new data were created or analyzed in this study. Data sharing is not applicable to this article.
